# Health among Swedish employees and financial situation, education, and managerial responsibility: A longitudinal study

**DOI:** 10.3109/03009734.2012.703254

**Published:** 2012-10-30

**Authors:** Roma Runeson, Eva Vingård, Erik Lampa, Kurt Wahlstedt

**Affiliations:** Occupational and Environmental Medicine, Akademiska Hospital, Ulleråkersvägen 40, SE-751 85 Uppsala, Sweden

**Keywords:** Education, financial situation, health, managerial responsibility

## Abstract

**Background.:**

The present study is part of a 3-year longitudinal study on work and health among employees in the public sector in Sweden. The aim was to study associations between self-rated health (SRH) and financial situation, education, and managerial responsibility.

**Methods.:**

Of the 9003 employees, 7533 answered the baseline questionnaires (84%). Altogether 9373 subjects received the follow-up questionnaire, and 6617 subjects responded (71%). In total 4240 completed the questionnaire on both occasions, and this group comprised the study population. SRH consisted of the response to a single question: ‘In general, would you say your health is excellent, very good, good, poor, or very poor?' The health was investigated in terms of the development of health status in the 3-year follow-up. The exposure factors were: financial situation, education, and managerial responsibility. Odds ratios were analysed using logistic regressions.

**Results.:**

Good financial situation and further education were predictors in maintaining good health and in avoiding poor health. The analysis also indicated the following determinants of sustained good SRH: having a good financial situation (OR 1.99 at baseline and OR 1.87 at follow-up), having a further education compared to lower education (OR 1.17 at baseline), and not having a worsening financial situation between baseline and follow-up (OR 0.53).

**Conclusion.:**

Financial situation and educational level were important factors that influence the subjective perception of health.

## Introduction

Health is a complex concept, and no generally accepted definition exists. The World Health Organization (WHO) first defined health in a utopian way as a state of complete physical, mental, and social well-being, and not merely the absence of disease or infirmity (1). In a Swedish review, health was defined in three concepts: as absence of illness, as a resource, and as strength (2). Another definition was given by Boorse, who stated that health is equivalent to normal functioning (3,4). With a background in the work of Whitbeck (5) and Pörn (6), for whom health meant having no disease and experiencing social well-being, the Swedish philosopher Nordenfelt (7) has characterized health as a person's ability, given standard circumstances, to achieve the vital goals and thus realize well-being.

Many factors are associated with health, such as age (8–11), sex (8–11), hereditary factors (12,13), social situation (14,15), smoking (16), overweight (17), physical, psycho-social, and organizational working conditions (8–11,18), sleep and recovery (19–26), and sense of coherence (27–31). Research on health is often performed by using a global measure of self-rated health from questionnaires. The person's estimation of his/her own health and the explanation factors as well as changes in health over time have been associated not only with diseases but also with personality traits, education, work situation, and social status (32). There are reports showing that self-reported health could be a good predictor of future morbidity and even mortality (9–11).

Recently it has been suggested that the status of the individual in his or her society is important for being in good health (33). According to Marmot, control, autonomy, and good social relations are important for health. Relationships between good health, high income, and high education have been debated, and there seems to be a positive health gradient (33). The aim of this study was to investigate the associations between self-rated health and some aspects of status, such as financial situation, education, and managerial responsibility among persons employed in the public sector in Sweden.

## Methods

The present study is part of the longitudinal study ‘Work and health in the public sector in Sweden', the HAKul study (34), which was launched in 1999–2000. The subjects came from six municipalities and four county councils. It was not possible to make a random selection of studied organizations because the participation was negotiated between employers and trade unions. However, we tried to make the selection of invited clusters as representative for municipalities and county councils in Sweden as possible. The selected clusters were from all parts of Sweden with different labour market conditions and represented almost all occupations in municipalities and county councils. Within the clusters all employees were invited to participate in the study. They worked in 120 occupations, the commonest being assistant nurses, home-based workers in elderly care, registered nurses, teachers, organizational managers, employees at child-care centres, mental care workers, administrative personnel, and doctors. At baseline the subjects had had no on-going absence from work for more than 3 months.

In total 9003 persons were invited to participate in the study, and 7533 responded (84%). They answered a questionnaire on individual factors, financial situation, managerial responsibility, education, and self-rated health (SRH). Three years later the same questionnaire was sent to all employed subjects 9373 in the same organizations, and 6617 responded (71%). In total 4539 completed the questionnaire on both occasions. Of these, 4240 responded to all questions on SRH, financial situation at first occasion (baseline), and at second occasion (follow-up), and to questions on education and managerial responsibility at baseline, and these comprised the study group. Data loss due to non-response to part of the questionnaire was 6.6%.

The outcome variable, *self-rated health* (SRH), was assessed by one question: ‘In general, how would you say your health is?' The answer alternatives were ‘excellent', ‘very good', ‘good', ‘poor', or ‘very poor'. Self-rated health was categorized into two categories: good (excellent, very good, and good), and poor (poor and very poor).

The changes in self-rated health were investigated in several ways: improved (poor at baseline and good at follow-up), worsening (good at baseline and poor at follow-up), sustained good (good at baseline and good at follow-up), and sustained poor (poor at baseline and poor at follow-up).

The exposure factors were: *financial situation*, divided into three categories: good, neither good nor strained, and strained. *The change in financial situation between baseline and follow-up*, divided into two categories: improved (from strained/neither good nor strained to good) and worsening (from good/neither good nor strained to strained); *education*, divided into two categories: further (university, college degree, and other higher educations) and non-academic (elementary school, vocational school, junior secondary education, and 2–4 years of secondary education); and *managerial responsibility*, divided into two categories: top managerial responsibility (high organizational and personnel responsibility) and other (having no or middle managerial responsibility).

Smoking was assessed by one question if a person was smoking daily (1 = yes, 0 = no), and 39 subjects (1%) did not answer this question. Body mass index (BMI) was classified according to international reference data (35), and 80 subjects (2%) did not give enough information to compute BMI.

### Statistical analysis

All statistical analyses were performed with SPSS (36). The differences between smoking, BMI, and the outcome variables were calculated by chi-square. Logistic regression analyses were performed. Three different models were performed for four different SRH outcomes (improved, worsening, sustained good, and sustained poor), using in model 1 the financial situation at baseline, in model 2 the financial situation at follow-up, and in model 3 the change in financial situation between baseline and follow-up. Additional independent variables were educational level and managerial responsibility, both assessed at baseline. The analyses were controlled for the minimum of factors: age, sex, smoking, and BMI. In the logistic regression analyses BMI was used as a continuous variable.

### Ethics

The study was approved by the ethics committee for the Karolinska Institute.

## Results

The majority of subjects had sustained good SRH (females 67% and males 73%). The smallest group was the one with improved SRH (female 7% and males 5%). There was a small difference in the mean age between the four groups. Smoking was more common in the groups with worsening and sustained poor SRH compared with the group with sustained good SRH (*P* < 0.001). BMI was associated with all four SRH groups (*P* < 0.01 for improved SRH and *P* < 0.001 for the other three groups) (Table I). Women comprised a majority of the study group (84% at baseline), and ages at baseline varied between 20 and 63 years with a mean value of 46 *±* 9 years. A total of 24% of the men and 27% of the women were smokers. At baseline, 9% of the men and 10% of the women were obese (BMI >29.9); and 44% of the men and 58% of the women had normal weight at baseline (BMI 18.5–24.9).

**Table I.** The characteristics of the study sample consisting of persons that responded at both baseline and follow-up (*n* (%) and mean ± SD).

	Self-rated health				
	Improved	Worsening	Sustained good	Sustained poor	Total
	277 (6)	556 (13)	2864 (68)	543 (13)	4240 (100)
Sex					
Female	241 (7)	474 (13)	2370 (67)	475 (13)	3560 (100)
Male	36 (5)	82 (12)	494 (73)	68 (10)	680 (100)
Age (mean ± SD)^a^	46 ± 9	48 ± 9	46 ± 9	49 ± 8	46 ± 9
Current smoker (*n*, %)	78 (7)	184 (17)^b^	657 (59)^b^	191 (17)^b^	1110 (100)
Body mass index (BMI)					
Underweight (>18.5)	4 (10)	4 (10)	28 (70)	4 (10)	40 (100)
Normal weight (18.5–24.9)	126 (5)	264 (11)	1706 (74)	232 (10)	2328 (100)
Overweight (25.0–29.9)	106 (8)	201 (14)	873 (63)	206 (15)	1386 (100)
Obesity (<29.9)	39 (9)	76 (19)	202 (50)	89 (22)	406 (100)
Financial situation at baseline					
Good	80 (4)	195 (11)	1398 (78)	131 (7)	1804 (100)
Neither/nor	143 (8)	250 (14)	1128 (63)	275 (15)	1789 (100)
Strained	61 (9)	111 (17)	338 (52)	137 (21)	647 (100)
Change in financial situation					
Improved	61 (7)	102 (12)	582 (70)	91 (11)	835 (100)
Unchanged	179 (6)	371 (13)	2025 (69)	362 (12)	2937 (100)
Worsening	37 (8)	83 (18)	257 (55)	90 (19)	468 (100)
Education at baseline					
Further	162 (7)	282 (12)	1697 (70)	268 (11)	2401 (100)
Non-academic	115 (6)	274 (15)	1167 (63)	275 (15)	1839 (100)
Managerial responsibility at baseline					
Top manager	57 (6)	109 (11)	673 (71)	109 (11)	948 (100)
Other^c^	220 (7)	447 (13)	2191 (67)	434 (13)	3292 (100)

^a^Presented as mean ± standard deviation (SD).

^b^Calculated by chi-square, *P* < 0.001.

^c^Other managerial (none and middle).

### Associations between the exposure factors and improved SRH

A good financial situation at baseline was negatively associated with an improved SRH (Table II). However, in this group, 88% had good, very good, or excellent health at baseline.

**Table II.** Improved self-rated health (SRH) during the 3-year follow-up related to exposures: financial situation, education, and managerial responsibility. Odds ratios (OR) and 95% confidence intervals (CI), *n* = 4240.

	Improved SRH		
	Model 1	Model 2	Model 3
Exposure	OR (95% CI)	OR (95% CI)	OR (95% CI)
Financial situation at baseline^a^			
Strained	1.30 (0.95–1.58)		
Good	0.55*** (0.41–0.74)		
Neither good nor strained	Ref		
Financial situation at follow-up			
Strained		1.08 (0.74–1.56)	
Good		0.80 (0.61–1.05)	
Neither good nor strained		Ref	
Change in financial situation^b^			
Worsening			1.30 (0.89–1.89)
Improved			1.23 (0.91–1.67)
Unchanged			Ref
Education^a^			
Further	1.22 (0.95–1.58)	1.17 (0.90–1.51)	1.12 (0.87–1.44)
Non-academic	Ref	Ref	Ref
Managerial responsibility^a^			
Managerial (top manager)	0.96 (0.70–1.30)	0.92 (0.68–1.25)	0.90 (0.66–1.22)
All others (none and middle)	Ref	Ref	Ref

Logistic regression models, adjusting for personal factors (age, sex, smoking, and BMI).

^a^Education and managerial at baseline.

^b^Change in financial situation between baseline and follow-up.

*P < 0.05; **P < 0.01; ***P < 0.001 by logistic regression.

Ref = reference category.

### Associations between the exposure factors and worsening SRH

A strained financial situation, at baseline or at follow-up, and worsening financial situation were positively associated with worsening SHR. On the other hand, having a good financial situation at baseline or at follow-up was negatively associated with worsening SRH. Having a further education was an under-risk for a worsening SRH (Table III).

**Table III.** Worsening of self-rated health (SRH) during the 3-year follow-up related to exposures: financial situation, education, and managerial responsibility. Odds ratios (OR) and 95% confidence intervals (CI), *n* = 4240.

	Worsening SRH		
	Model 1	Model 2	Model 3
Exposure	OR (95% CI)	OR (95% CI)	OR (95% CI)
Financial situation at baseline^a^			
Strained	1.32* (1.03–1.70)		
Good	0.80* (0.65–0.98)		
Neither good nor strained	Ref		
Financial situation at follow-up			
Strained		1.56** (1.20–2.02)	
Good		0.71** (0.58–0.87)	
Neither good nor strained		Ref	
Change in financial situation^b^			
Worsening			1.54** (1.18–2.02)
Improved			0.99 (0.78–1.25)
Unchanged			Ref
Education^a^			
Further	0.76** (0.63–0.92)	0.80* (0.66–0.96)	0.74** (0.61–0.89)
Non-academic	Ref	Ref	Ref
Managerial responsibility^a^			
Managerial (top manager)	0.89 (0.71–1.12)	0.90 (0.72–1.13)	0.87 (0.69–1.09)
All others (none and middle)	Ref	Ref	Ref

Logistic regression models, adjusting for personal factors (age, sex, smoking, and BMI).

^a^Education and managerial at baseline.

^b^Change in financial situation between baseline and follow-up.

*P < 0.05; **P < 0.01; ***P < 0.001 by logistic regression.

Ref = reference category.

### Associations between the exposure factors and sustained good SRH

A good financial situation, at baseline or at follow-up, was positively associated with sustained good SRH after 3 years of follow-up. A strained financial situation, at baseline or at follow-up, and a worsening in financial situation between baseline and follow-up were negatively associated with sustained good SRH. Having a further education was positively associated with sustained good SRH (Table IV).

**Table IV.** Sustained good self-rated health (SRH) during the 3-year follow-up related to exposures: financial situation, education, and managerial responsibility. Odds ratios (OR) and 95% confidence intervals (CI), *n* = 4240.

	Sustained good SRH		
	Model 1	Model 2	Model 3
Exposure	OR (95% CI)	OR (95% CI)	OR (95% CI)
Financial situation at baseline^a^			
Strained	0.59*** (0.48–0.71)		
Good	1.99*** (1.70–2.32)		
Neither good nor strained	Ref		
Financial situation at follow-up			
Strained		0.52*** (0.42–0.64)	
Good		1.87*** (1.61–2.17)	
Neither good nor strained		Ref	
Change in financial situation^b^			
Worsening			0.53*** (0.43–0.65)
Improved			1.00 (0.84–1.19)
Unchanged			Ref
Education^a^			
Further	1.17* (1.02–1.35)	1.14 (0.99–1.31)	1.29*** (1.13–1.48)
Non-academic	Ref	Ref	Ref
Managerial responsibility^a^			
Managerial (top manager)	1.08 (0.92–1.28)	1.09 (0.92–1.29)	1.16 (0.99–1.37)
All others (none and middle)	Ref	Ref	Ref

Logistic regression models, adjusting for personal factors (age, sex, smoking, and BMI).

^a^Education and managerial at baseline.

^b^Change in financial situation between baseline and follow-up.

*P < 0.05; **P < 0.01; ***P < 0.001 by logistic regression.

Ref = reference category.

### Associations between the exposure factors and sustained poor SRH

A good financial situation, at baseline or at follow-up, was negatively associated with sustained poor SRH after 3 years of follow-up. But having a strained financial situation, at baseline or at follow-up, and a worsening in financial situation between baseline and follow-up were positively associated with sustained poor SRH (Table V).

**Table V.** Sustained poor self-rated health (SRH) during the 3-year follow-up related to exposures: financial situation, education, and managerial responsibility. Odds ratios (OR) and 95% confidence intervals (CI), *n* = 4240.

	Sustained poor SRH		
	Model 1	Model 2	Model 3
Exposure	OR (95% CI)	OR (95% CI)	OR (95% CI)
Financial situation at baseline^a^			
Strained	1.67*** (1.31–2.13)		
Good	0.43*** (0.34–0.54)		
Neither good nor strained	Ref		
Financial situation at follow-up			
Strained		1.88*** (1.46–2.43)	
Good		0.46*** (0.37–0.57)	
Neither good nor strained		Ref	
Change in financial situation^b^			
Worsening			1.82*** (1.39–2.38)
Improved			0.90 (0.70–1.16)
Unchanged			Ref
Education^a^			
Further	0.84 (0.69–1.01)	0.87 (0.72–1.06)	0.75** (0.62–0.91)
Non-academic	Ref	Ref	Ref
Managerial responsibility^a^			
Managerial (top manager)	1.01 (0.80–1.28)	1.01 (0.80–1.28)	0.93 (0.74–1.17)
All others (none and middle)	Ref	Ref	Ref

Logistic regression models, adjusting for personal factors (age, sex, smoking, and BMI).

^a^Education and managerial at baseline.

^b^Change in financial situation between baseline and follow-up.

*P < 0.05; **P < 0.01; ***P < 0.001 by logistic regression.

Ref = reference category.

## Discussion

This 3-year longitudinal study investigated associations between self-rated health among working women and men from the public sector in Sweden, and educational level, financial situation, and managerial responsibility.

Good financial situation and further education were predictors in maintaining good health and in avoiding poor health. On the other hand, having a strained financial situation increased the risks for getting or retaining poor health. A worsened financial situation was negatively associated with sustaining good SRH, and a good financial situation was preventive of getting poor health. The results do not differ from other countries where there is clearly greater inequality in income (33,37).

Wang et al. (38) found, in accordance with our study, that income had strong associations with self-reported fair or poor health in middle-aged and elderly Japanese men and women. Contrary to our findings, in a Russian cross-sectional survey, education was inversely related to self-rated health, but the major finding was that poor health status in Russia is related to dysfunction of social structures, socio-economic deprivation, and lack of perceived control (39). Sen (40) pointed out that lacking financial means may be a cause of inability to pursue well-being.

According to Sen (40) a person can have the financial means to live well but can still generate some deprivation, possibly due to a lack of adequate personal abilities to live well. In our study a good financial situation did not help in changing from poor to good health. When health is deteriorated it might not always be possible to reverse it, even by having good finances. In a 16-year follow-up of a randomized normal population, Ahnquist et al. (41) found that financial stress (i.e. for example not being able to pay bills and lack of cash reserves) was associated with effects on health for both men and women. However, they found that low income was not associated with poor health among women, and among men it was associated with psychological stress. In the present study, where a majority was women and all were employed and they were basically a healthy working population, the associations between a strained financial situation both at baseline and at follow-up was a risk for losing good health and maintaining poor health.

High education was positively associated with good health in accordance with other studies. Marmot (42) has pointed out that educational level is important for health. In our study, financial situation was also a predictor of good health even when education was included in the regression model. This is contrary to Marmot's finding (42), where income level was no longer significant when educational level was introduced in the model. This might reflect differences between men and women, since we studied mainly women and Marmot studied mainly men. It is possible that educational background is associated with high income to a higher degree among men in England than among women employed in the Swedish public sector with relatively low and equal salary levels.

Good management has proved to influence health among subordinates in a positive way (43). In this study there were no associations between managerial responsibility and self-rated health. In contrast to Marmot's findings, in this study occupational status was not a predictor of good health. This might reflect cultural differences between Sweden and the UK regarding the status of managers. However, our material was collected only among employees working in the public sector, which makes comparison between the findings more difficult. The health of executive women was studied in a previous study (44), and it showed that women in executive positions reported greater life satisfaction and better health than non-executive working women. These results were not replicated in our study.

One limitation of this study is that this was not a random sample from a normal population. It was confined to the working population, mainly women, in the public sector, which limits generalizability. A thorough analysis showed no major differences between participants and non-participants, with respect to sex, education, financial situation, and managerial responsibility measured at baseline. The differences that were found concerned age and self-reported health. The non-participants were younger and their health was inferior compared with the study population (a suspected healthy worker effect). There was an odd finding in our study which was that an improved financial situation was neither associated with maintaining good health nor retaining poor health; however, the groups with a major change of financial situation were relatively small which reduces generalizability. Furthermore, a good financial situation at baseline was associated with a lower possibility of a change from poor to good health; this was also a very small group which makes interpretation difficult. In this group there was a small margin for improvement of health since the great majority (88%) had good, very good, or excellent health.

No objective measures of health were used in this study. However, there are recent reports showing that self-reported health could be a better predictor of future health outcome than clinically diagnosed health (41,45). These results are in accordance with earlier findings (9,11) which support the use of self-reported data on health in the present study. The findings in this study support the existence of influence of financial situation on health (33,37) in the presumably healthy and currently working Swedish employees from the public sector.

## Conclusion

The main findings of this study were that the financial situation and educational level were important factors that influence the subjective perception of health.
